# PTBP1 and Cancer: From RNA Regulation to Therapeutic Potential

**DOI:** 10.1111/jcmm.70675

**Published:** 2025-06-28

**Authors:** Chengdong Ji, Jing Zhu, Xiaochen Hou, Chen Zhou, Jiumei Zhao, Xiang Zheng, Yu Tang

**Affiliations:** ^1^ Pathology Department The Ninth People's Hospital of Suzhou Suzhou China; ^2^ Yunan Key Laboratory of Breast Cancer Precision Medicine, School of Biomedical Engineering Kunming Medical University Kunming China; ^3^ The Third Affiliated Hospital of Kunming Medical University Kunming Medical University Kunming China; ^4^ Department of Laboratory, Chongqing Nanchuan District People's Hospital Chongqing Medical University Chongqing China; ^5^ Department of Genetics Zunyi Medical University Zunyi Guizhou China

**Keywords:** alternative splicing, PTBP1, RNA modification, targeted therapy, tumour translation

## Abstract

Polypyrimidine tract binding protein 1 (PTBP1) belongs to the heterogeneous nuclear ribonucleoprotein (hnRNP) family and is a widely studied RNA‐binding protein involved in regulating mRNA splicing, stability, localization, and translation. In addition, PTBP1 can participate in various non‐coding RNA action processes and affect tumorigenesis and progression. In tumour therapy, PTBP1 may act as a key factor influencing targeted drug targets. Therefore, in this paper, we reviewed the structure and function of PTBP1, its role in various tumours, and key mechanisms of action. Moreover, we discuss the difficulties and challenges faced in PTBP1 research and the clinical translational potential of PTBP1 research and summarise the future research direction of PTBP1 to provide new ideas for basic research and new directions for precision treatment of tumours in the clinic.

## 1 Introduction

The annual global incidence of cancer is still on the rise, and cancer remains a significant health challenge worldwide [1]. According to the latest data, it is estimated that there will be 2,041,910 new cancer diagnoses (5600 per day) and 618,120 cancer deaths in the United States. Furthermore, by 2025, there will be approximately 59,080 new cases of ductal carcinoma in situ in women and 107,240 new cases of cutaneous melanoma in situ [2]. At present, the main treatment methods for cancer patients include surgical resection, radiotherapy, chemotherapy and small molecule targeted drug therapy. Among them, targeted therapy can be applied to different tumour subtypes. Unlike chemotherapy that kills cancer cells, targeted therapy targets identified carcinogenic sites at the cellular and molecular level, thereby preventing the replication and proliferation of cancer cells while limiting damage to normal cells. However, targeted therapy also has its limitations. At present, there are few therapeutic targets for targeted therapy, and drug resistance may occur during the treatment process [3, 4, 5]. Therefore, current research is needed to find new therapeutic targets for tumours and explore the mechanism of tumour resistance during targeted therapy.

There are approximately 1914 human RNA binding proteins (RBPs), accounting for 7.5% of the coding genes [6, 7, 8]. RBPs are highly conserved and play a key role in maintaining the homology of gene expression [9, 10]. It has been found that RBPs are involved in various critical cellular processes, such as transport, localization, development, differentiation, and metabolism [11, 12]. In addition, RBPs are involved in post‐transcriptional regulation, regulating transcript formation and function, and maintaining cellular homeostasis [13]. Mechanistically, RBPs regulate variable splicing, polyadenylation, mRNA stability, localization, and translation by interacting with RNA substrates [14, 15]. Polypyrimidine tract binding protein 1 (PTBP1) belongs to the family of nuclear heterogeneous ribosomal proteins (hnRNPs) and is an RNA‐binding protein closely related to RNA modification [16]. PTBP1 belongs to the PTB family, which also contains PTBP2 and PTBP3 [17]. PTBP1 is expressed in almost all cell types and regulates a wide range of biological processes, whereas PTBP2 is expressed only in the nervous system, and PTBP3 is found mainly in immune cells [18, 19, 20, 21]. These three homologous proteins share more than 70% sequence homology at the protein level, and all have four RNA recognition motifs (RRMs) for RNA binding [22, 23]. Studies have shown that PTBP1 plays an important regulatory role in mRNA modification and metabolism. The most widespread role of PTBP1 is to participate in the Alternative Splicing process (AS) of heterogeneous nuclear RNA (hnRNA) as a splicing factor. PTBP1 can affect multiple aspects such as AS cell cycle, differentiation, metabolism, movement, and immunity by regulating AS [24]. Moreover, PTBP1 regulates the stability of mRNAs and affects their replication, transport, translation, and cytoplasmic localization through nuclear‐cytoplasmic shuttling and polyadenylation [25, 26, 27, 28].

Cancer development is a multigene, multistep, multistage process involving multiple genes, and cancers often have aberrant expression of PTBP1, a process that is closely related to cancer development [29]. An increasing number of studies have shown that PTBP1‐mediated mRNA alterations have essential functions in tumours, including colorectal cancer [30], hepatocellular carcinoma [31], breast cancer [32], and oesophageal squamous cell carcinoma [33]. In particular, in cancer cells, glycolysis is the most critical process that PTBP1 is involved in regulating, and PTBP1 promotes the expression of the M2 isoform of pyruvate kinase (PKM2) while decreasing the expression of PKM1, which mainly leads to a shift from oxidative phosphorylation metabolism to glycolytic metabolism and affects tumorigenesis in cancer cells [34]. PTBP1 regulates apoptosis, proliferation, migration, and invasion through different pathways and molecules [35, 36, 37, 38]. Therefore, by revealing the expression mechanism of PTBP1 and the interaction between PTBP1 and its target RNAs, new ideas or methods can be provided to find new targets for cancer therapy.

In summary, based on the direct link between PTBP1 and tumour progression, this review provides a comprehensive analysis of the relevant studies on PTBP1 and tumours in recent years to elucidate the role of PTBP1 in tumours, the mechanism, and its clinical significance. It aims to provide a theoretical basis for basic research on the role of PTBP1 in tumours and clinical targeting therapies.

### 1.1 PTBP1 Gene Structure

The human PTBP1 gene is located on chromosome 19p13.3 and encodes a protein, PTBP1, consisting of 531 amino acids [39] containing an N‐terminal nuclear localization signal (NLS) and four RRM‐type RNA binding domains (RBDs) containing RBD1, RBD2, RBD3, and RBD4, which are responsible for acting as RNA binding structural domains connected via flexible junction peptides, which typically bind to pyrimidine‐rich regions of the RNA sequence [23, 40]. BRDs consist of 90 bases with typical βαββαββαβ RRM topology and an α/β tertiary fold and are connected by three linker regions. In particular, RBD2 and RBD3 contain an additional 5thβ strand, and the four RRMs are at variance with the conserved RRM sequences that differ from each other, usually manifested by the absence of aromatic side chains [41, 42, 43]. These specific sequences allow RBD1 and RBD2 to bind short sequences rich in UC bases and RBD3 and RBD4 to bind long sequences rich in UC bases [44]. Moreover, it has been shown that each RBD‐binding RNA has different binding specificities; for example, RBD1 and RBD3 of PTBP1 are indispensable for regulating downstream target gene HIF‐1α mRNA stability [45]. Whereas RBD2 is mainly involved in protein binding [46], RBD3 is a determinant of PTBP1 RNA binding specificity [47].

**FIGURE 1:**
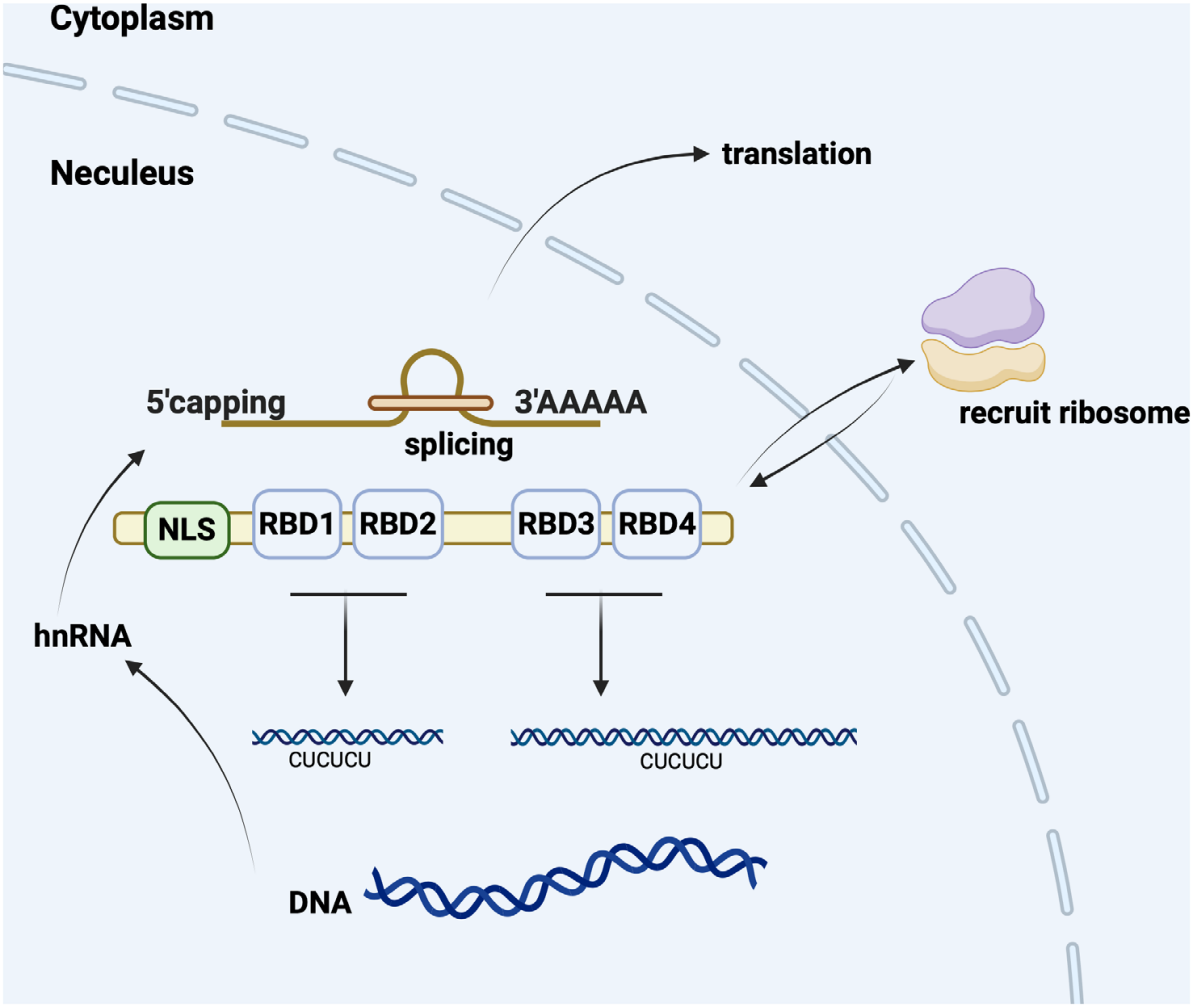
Structure and biological function of PTBP1. PTBP1 is an RNA‐binding protein with a significant nuclear localization function and is able to freely enter and exit the nucleus via the N‐terminal nuclear localization signal (NLS) and four RNA‐binding domains (RBDs). After the DNA is transcribed into precursor mRNA, PTBP1 binds to 15–25 pyrimidine base sequences rich in U and C and is involved in selective mRNA splicing, 5′ capping, 3′ polyadenylation, termination of protein translation, and RNA degradation.

Taken together, PTBP1 binds RNAs containing complex sequences and is involved in RNA AS, 5′ capping, 3′ polyadenylation, termination of protein translation, and RNA degradation. Thus, it plays an important role in modifying RNA structure and ensuring the diversity of RNA transcripts and their functions (Figure 1).

### 1.2 The Molecular Biological Function of PTBP1

PTBP1 is a regulatory factor involved in post‐transcriptional gene expression regulation, such as RNA variable splicing, polyadenylation, nucleoplasmic shuttling, mRNA stability, translation, and localisation. It has a variety of metabolism‐related functions [25, 26, 27, 28]. Among them, PTBP1 most widely regulates RNA.

**FIGURE 2:**
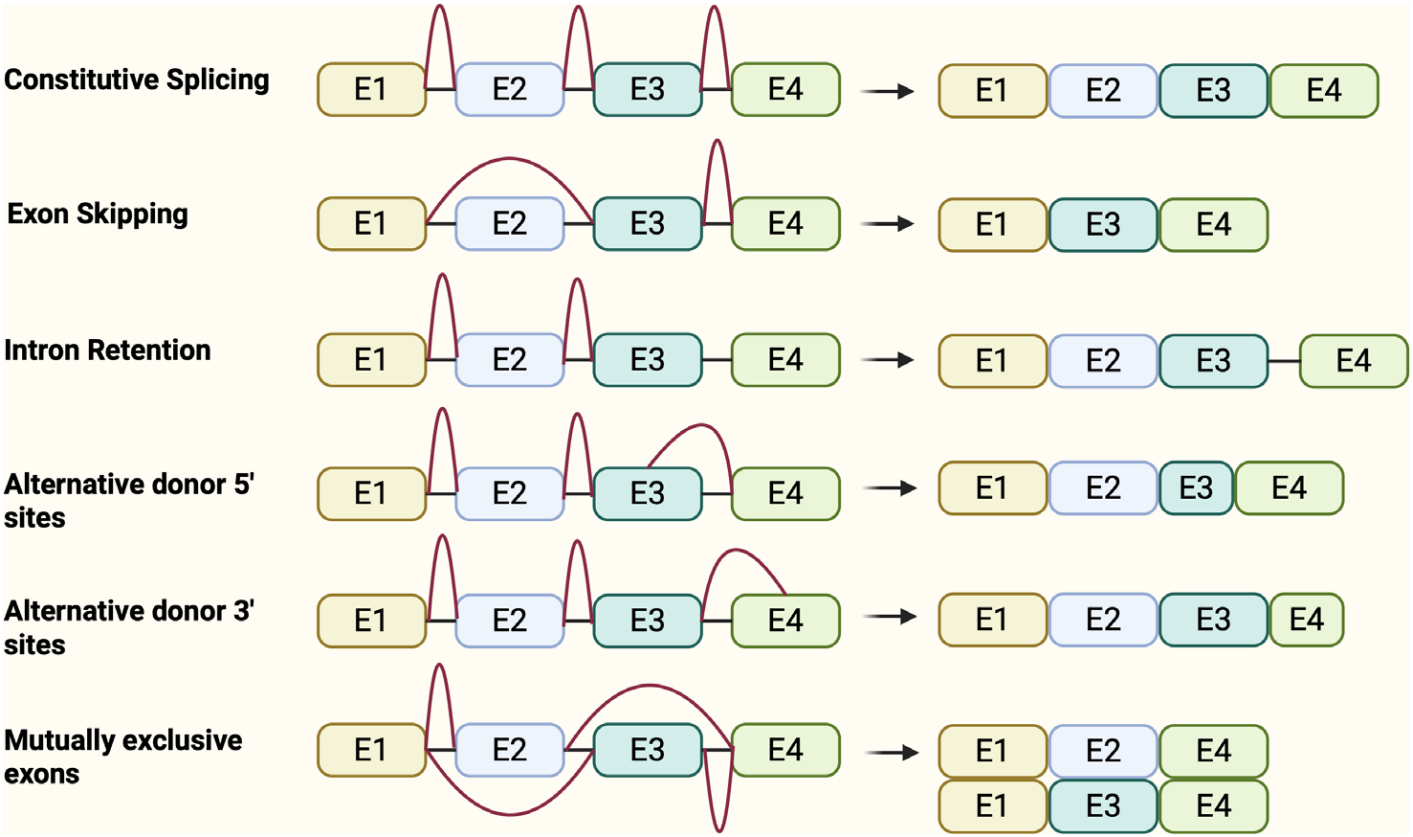
Variable splicing event types. On the left are the different pre‐mRNA transcripts, the exon is E, the intron is the black connecting line, and the red line represents the splicing event connecting the exon. The figure on the right shows mRNA transcripts processed by different splicing events.

The hnRNAs transcribed from eukaryotic genes contain alternatively linked introns and exons [48, 49]. Typically, hnRNA becomes a mature mRNA after the intronic sequence is eliminated and translated into a corresponding polypeptide chain [50]. However, exons involved in splicing can be spliced out of the order of linear distribution within the genome in which they are located, and introns can be incompletely excised, resulting in variable splicing, and variable splicing results in the production of different mature mRNAs from the same hnRNA [51]. Variable splicing mainly includes six types: CS, ES, A3SS, A5SS, IR and MEE [52] (Figure 2). Selective splicing of exons or introns is regulated by various cis‐acting elements and transacting splicing factors [53]. Variable splicing increases the diversity of transcripts and the complexity of the proteome. Cont variable splicing is involved in all stages of cell growth and differentiation and is also closely associated with the development of several human diseases [54, 55, 56, 57]. PTBP1 can generate different splicing variants by splicing precursor mRNAs of downstream target genes, which may lead to different cellular phenotypes with different travelling function protein products [58]. PTBP1 is not an absolute splicing repressor or splicing activator, and whether it acts as a repressor or an activator of selective splicing depends on the location of the binding site of PTBP1 to the exon [53]. For example, PTBP1 promotes exon retention when it binds to exons or flanking intronic sequences around alternate exons and prevents exon splicing when it binds to polypyrimidine sequences within the 3′ splice site and represses exon selection [59]. Moreover, under most normal conditions, PTBP1 is expressed in the nucleus, binds introns or exons of polypyrimidines in hnRNA, and can negatively regulate splice sites on both sides by wrapping exons so that they do not bind to the spliceosome and cause exon jumping, leading to nonsense‐mediated decay and early termination of codon insertion [53, 60, 61]. Thus, PTBP1‐mediated exon skipping interferes with exon biological processes, affects splice site selection, and limits gene overexpression [62]. Taken together, PTBP1 affects multiple biological processes through variable splicing processes (Table 1).

**TABLE 1:** PTBP1 is involved in various tumour‐related biological processes.

Target gene	Mechanism	Biological process	References
*PKM*	PKM1/PKM2 subtype conversion promotes glucose metabolism in cancer cells	Cell metabolism	[62]
*BAF45d*	Jumping 6A exons regulate multiple key proteins in cellular metabolic pathways	Cell metabolism	[63]
*BCL‐X*	Promote the expression of exon 2 of BCL‐X and generate the proapoptotic subtype BCL‐XS	Cell cycle and apoptosis	[64]
*BIM*	Jumping exon 3 inhibits apoptosis	Cell cycle and apoptosis	[65]
*RIPK1*	Jumping deleterious recessive exons regulate apoptotic or necrotic cell death pathways	Cell cycle and apoptosis	[66]
*USP5*	Jump exon 15, generate short subtype USP5‐2, promote cell migration and invasion	Cell movement	[67]
*RTN4*	Jumping exon 3 reduces cell adhesion to fibronectin and hyalbenin	Cell movement	[68]
*Axl*	Jump exon 10 to form Axl‐S subtype; It leads to the phosphorylation of ERK and AKT, and promotes the migration and invasion of liver cancer cells	Cell movement	[69]
*Dab1*	Jumping exons 7 and 8 attenuated signal transduction and inhibited late neuron migration	Cell movement	[70]
*Mena*	The retention of Mena11a exon promotes the formation of filamentous pseudopodia	Cell movement	[71]

Moreover, PTBP1 also has important regulatory functions for the stability, localization, translation of mRNA and non‐coding RNA. Research has found that PTBP1 is involved in the transport and localization of mRNA within cells, ensuring that mRNA is translated at the appropriate time and place. For instance, PTBP1 helps transport mRNA to specific cellular regions through its interaction with the cytoskeleton, which is particularly significant in the formation of synapses in nerve cells. PTBP1 can regulate the stability of mRNA and protein translation by binding to the 3′‐untranslated region (UTR) or 5′‐untranslated region (UTR) of the target gene mRNA [59]. Some gene mRNAs contain internal ribosome entry sites (IRES) on the 5′‐UTR, and PTBP1 can also promote IRES‐mediated protein translation by binding to the 5′‐UTR [72]. Furthermore, PTBP1 plays a regulatory role in the biosynthesis of miRNA. By regulating the splicing and maturation of precursor miRNA, PTBP1 affects the expression level of miRNA, which is crucial for the regulation of gene expression in cells [73]. Studies have found that PTBP1 combines miR‐137 and miR‐206 and promotes carcinogenesis by disrupting the characteristics of normal tissues [74]. The interaction with miR‐133b and miR‐1 can enhance the specific metabolism of cancer cells (such as glycolysis), providing energy support for their proliferation [75]. miR‐145 hinders tumorigenesis by interfering with the Warburg effect (inhibiting glycolytic dependence), suggesting that PTBP1 may antagonise its function to maintain the metabolic dominance of cancer cells [76]. Furthermore, the combination of PTBP1 with Linc‐ROR and miR‐124 can induce the resistance of cancer cells to gemcitabine (a chemotherapy drug), which may be achieved by activating survival signals or inhibiting the drug target pathway [77]. As a carcinogenic circular RNA, CircSMARCAS may directly promote tumour formation through PTBP1‐mediated RNA stability regulation or signal transduction [78] (Table 2). These findings reveal the core role of PTBP1 in cancer metabolic reprogramming, therapeutic resistance and malignant transformation by dynamically binding to different RNAs, providing a theoretical basis for novel therapeutic strategies targeting the PTBP1‐RNA interaction network.

**TABLE 2:** In cancer, PTBP1 binds to LncRNA, CircRNA and miRNA.

RNA type	Function	References
miR137 and miR206	Negative effects related to tissue characteristics and carcinogenesis	[74]
miR‐133b and miR‐1	Positive regulation of cancer‐specific energy metabolism	[75]
miR‐145	Perturbation of the Warburg effect and inhibition of carcinogenesis	[76]
Linc‐ROR、miR‐124	Resistance to gemcitabine	[77]
CircSMARCA5	Oncogenesis	[78]

In summary, PTBP1, as a multifunctional RNA‐binding protein, not only plays an essential role in mRNA splicing, maturation, translation, and stabilisation but also has significant biological significance in intracellular mRNA localization and microRNA biosynthesis. Therefore, the study of PTBP1 will help us understand how cells adapt to different physiological needs and pathological states by regulating gene expression.

### 1.3 PTBP1 Abnormalities in Cancer

Tumours are complex and heterogeneous diseases whose pathogenesis is difficult to determine. They are always accompanied by genetic mutations that disrupt the homeostasis of oncogenic or tumour‐suppressive signalling pathways [79, 80]. PTBP1 is critical for regulating various important cellular processes, such as RNA variable splicing, transport, localisation, stability [25, 26, 27, 28], and so on (Table 3).

**TABLE 3:** Roles of PTBP1 in various cancers.

Type of cancer	Mechanism	Function	References
Colorectal cancer	The transformation from PKM1 to PKM2 is achieved through selective shearing	Promote the Warburg effect of tumour cells	[34]
	Autophagy is activated by stabilising BECN1 mRNA	Promote tumour progression	[37]
	Regulate the expression of key apoptotic proteins Bax and Bcl2	Inhibit the apoptosis of cancer cells	[30]
	circSCP2 regulates the miR‐92a‐1‐5p/IGF2BP1 pathway and promotes the interaction between PTBP1 and IGF2BP1	Promote the metastasis of colorectal cancer	[81]
Liver cancer	The tumour suppressor FGFR2‐IIIB is transformed into the oncogenic FGFR2‐IIIC subtype by regulating the selective splicing of FGFR2	Promote the progression of hepatocellular carcinoma	[82]
	By promoting the transport of FASN mRNA, a key gene for fatty acid synthesis, to the cytoplasm to facilitate its translation, it regulates lipid metabolism in hepatoma cells and thereby inhibits oxidative stress	Promote the progression of liver cancer	[83]
Lung cancer	Promote the transformation of PKM1 into PKM2	Promote the Warburg effect of lung cancer cells	[84]
	By binding to the flanking introns of circGLIS3, it promotes the generation of the oncogenic factor circGLIS3	Promote the proliferation, migration and invasion of tumour cells and enhance the progression of non‐small cell lung cancer	[85]
	PTBP1 can bind to AXL mRNA through RRM1, causing its instability to regulate AXL expression	Promote the progression of lung cancer	[59]
Gastric cancer	Negatively regulate the expression levels of RAGE and HMGB1	Promote the glycolysis, proliferation and migration abilities of gastric cancer cells	[86]
Glioma	Regulate the variable splicing of ITSN1	Promote the malignant progression of tumours	[87]
Breast cancer	By activating the PTEN/Akt pathway and autophagy	Maintain the growth and malignant characteristics of cancer cells	[88]
Bladder cancer	Promote the splicing of carcinogenic mutations	Promote cell proliferation, migration and invasion both in vitro and in vivo	[76, 89]
Prostate cancer	By interacting with RALY, it jointly regulates the selective splicing of DNA methyltransferase DNMT3B	Activate the pathways related to promoting survival or DNA repair, and ultimately enhance the resistance of cancer cells to radiotherapy	[90]

Studies have shown that PTBP1 abnormalities can lead to tumours. For example, in colorectal cancer, PTBP1 can convert PKM1 to PKM2 through selective shearing, promoting the Warburg effect in tumour cells [34]. In terms of regulating the mode of cell death, PTBP1 can participate in the regulation of autophagy and apoptosis in colorectal cancer cells through the regulation of downstream target genes, and it was found that PTBP1 activates autophagy by stabilising BECN1 mRNA to promote tumour progression [37]. Studies have found that colorectal cancer cells transfer circular RNA circSCP2 through exosomes. On the one hand, it adsorbs miR‐92a‐1‐5p through sponges to relieve its inhibitory effect on downstream target genes. On the other hand, it interacts with RNA‐binding protein PTBP1 to stabilise the oncogenic factor IGF2BP1. Thereby synergistically activating tumour metabolism and metastasis‐related pathways, and ultimately promoting the metastasis of colorectal cancer [81]. Moreover, PTBP1 can inhibit apoptosis in colorectal cancer cells by regulating the expression of apoptosis key proteins Bax and Bcl2 [30]. PTBP1 converts oncogenic FGFR2‐IIIb to pro‐carcinogenic FGFR2‐IIIc isoforms by modulating the selective splicing of FGFR2, which ultimately promotes hepatocellular carcinoma development [82]. Furthermore, PTBP1 is also involved in redox homeostasis in hepatocellular carcinoma cells. It was found that PTBP1 regulates lipid metabolism in hepatocellular carcinoma cells and thus inhibits oxidative stress and promotes hepatocellular carcinoma development by promoting the transport of the key gene for fatty acid synthesis, FASN mRNA, into the cytoplasm to facilitate its translation [83]. In lung cancer, PTBP1 can promote the Warburg effect in lung cancer cells by selectively splicing and promoting the conversion of PKM1 to PKM2, and circRNA EPB41L2 can play an anti‐tumour role by promoting the ubiquitination degradation of PTBP1 [84]. Moreover, PTBP1 can play a pro‐oncogenic role in lung cancer by promoting the cyclization of circular RNAs (circRNAs) with pro‐oncogenic function. It has been found that PTBP1 binds to the flanking intron of circGLIS3 to promote the production of the oncogenic factor circGLIS3, which promotes tumour cell proliferation, migration, and invasion and enhances the progression of non‐small cell lung cancer [85]. In addition, PTBP1 can also play a pro‐lung cancer role by regulating the stability of mRNA of downstream target genes, and PTBP1 can promote the progression of lung cancer through the binding of RRM1 to AXL mRNA, resulting in its unstable regulation of AXL expression [59]. Some studies have revealed the critical role of PTBP1 in the occurrence and development of gastric cancer. Some patients with gastric cancer and diabetes mellitus were found to have elevated expression of receptors for advanced glycation end products (RAGE) and high mobility group protein B1 (HMGB1) [86]. PTBP1 negatively regulates this process, and reducing the expression levels of RAGE and HMGB1 can inhibit the glycolysis, proliferation, and migration ability of gastric cancer cells [86]. It was found that down‐regulation of PTBP1 expression could attenuate the migration and invasion ability of gastric cancer cells, and PTBP1 could reduce the infiltration of immune cells in gastric cancer tissues and inhibit the immune function of the patients, thus further establishing the RBP model related to the prognosis of gastric cancer patients [91]. 26S proteasome non‐ATPase regulatory subunit 14, a deubiquitinating enzyme, is highly expressed in gastric cancer tissues, and it co‐localises and endogenously interacts with PTBP1, which can promote gastric cancer development by stabilising the molecular structure domain of PTBP1 [92]. Another study found that the knockdown of PTBP1 significantly inhibited the proliferation, migration, and invasion of gastric cancer cells in vitro and that PTBP1 could maintain the tumorigenic activity and stem cell properties of gastric cancer in vitro and in vivo [93]. PTBP1 is the first RNA‐binding protein reported to affect glioma behaviour, and the two are highly correlated. On the one hand, PTBP1 is aberrantly overexpressed in gliomas, indicating poor grading and prognosis. On the contrary, gliomas are sensitive to PTBP1 expression [87]; depletion of PTBP1 dramatically restricts their proliferation, migration, and adhesion [94, 95, 96]. For example, PTBP1 promotes malignant tumour progression by regulating variable splicing of ITSN1 [87]. In breast cancer on aberrant splicing by protein–protein interaction analysis, PTBP1 was identified as a central gene associated with brain metastasis of breast cancer [97]. Therefore, PTBP1 may be a new biomarker for breast cancer and a new target for therapy. Studies have shown that PTBP1 is involved in breast carcinogenesis and maintains the growth and malignant characteristics of cancer cells through activation of the PTEN/Akt pathway and autophagy [88] and that PTBP1 knockdown significantly inhibits cancer cell growth and invasion and increases oxygen consumption [98]. Studies have shown that in bladder cancer, PTBP1 promotes bladder cancer cell survival, facilitates splicing of oncogenic mutations, and promotes cell proliferation, migration, and invasion in vitro and in vivo [76, 89]. Furthermore, research has found that in prostate cancer, the RNA‐binding protein PTBP1 interacts with another RNA‐binding factor RALY to jointly regulate the selective splicing of DNA methyltransferase DNMT3B (promoting the generation of specific splicing variants), leading to abnormal DNA methylation patterns and thereby activating survival or DNA repair related pathways. Ultimately, enhance the resistance of cancer cells to radiotherapy [90].

In summary, PTBP1 plays multiple roles in tumorigenesis and development, and its properties as an RNA‐binding protein enable it to influence the biological behaviour of tumour cells by regulating gene splicing and expression. Further studies on PTBP1 function may provide new targets and strategies for tumour diagnosis and treatment in the future. By gaining a deeper understanding of the specific roles of PTBP1 in different tumour types, tumour biology can be better understood, thus promoting the development of precision‐targeted medicine.

### 1.4 The Molecular Mechanisms Underlying PTBP1 Roles in Cancer Progression

PTBP1 regulates tumour progression through multiple mechanisms, including variable splicing, stability, subcellular localization, and translation, and the diversity of these mechanisms makes the role of PTBP1 in the tumour microenvironment increasingly complex.

## 2 Alternative Splicing

**FIGURE 3:**
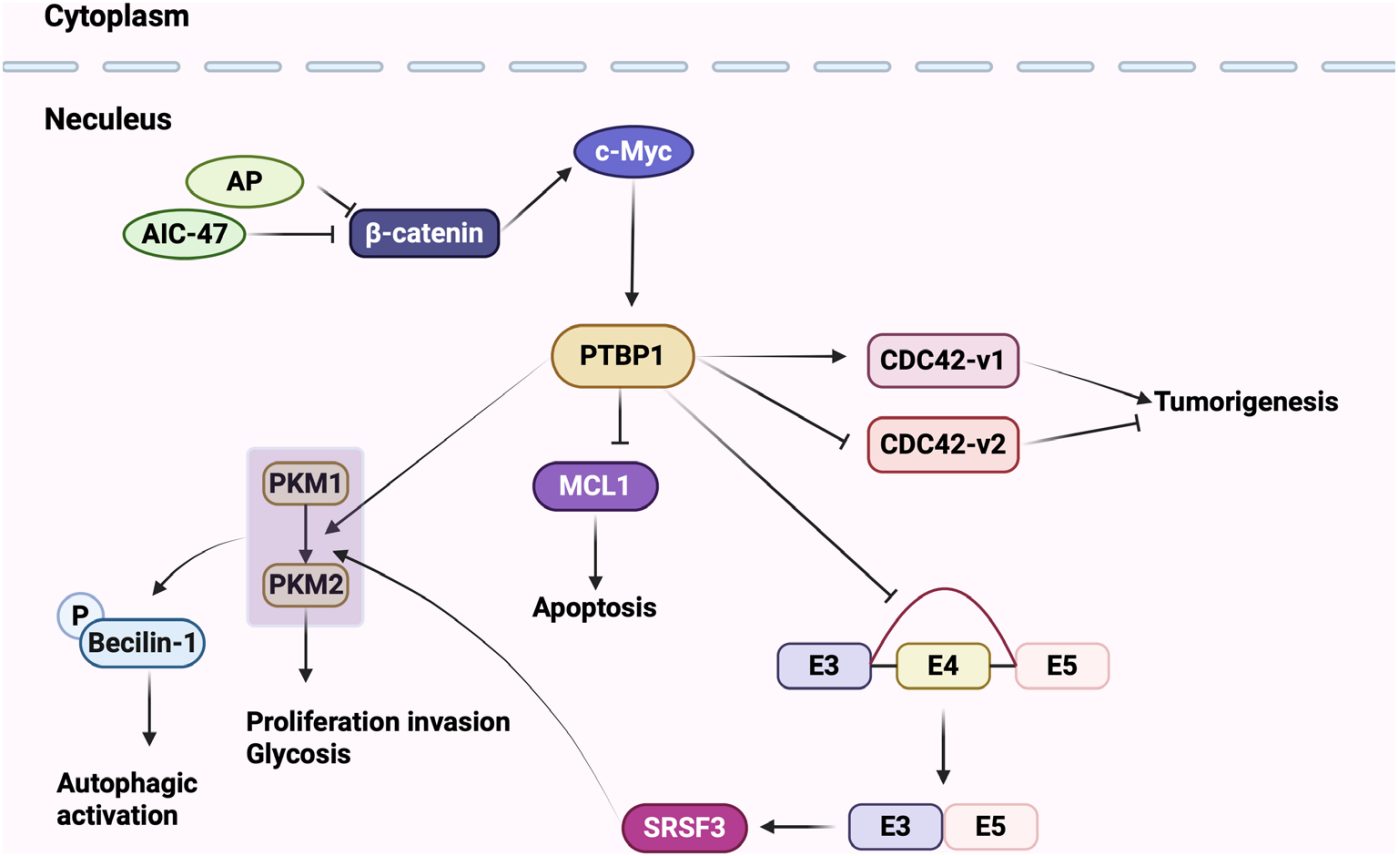
Functions and regulatory roles of PTBP1 in cancer cells.

Variable splicing is an important post‐transcriptional regulatory mechanism contributing to transcript and protein diversity [99]. The main types of aberrant splicing in tumours include: constitutive splicing, exon skipping or inclusion, variable 5′ splice sites, variable 3′ splice sites, intron retention, and exon mutual exclusion [100]. Aberrant PTBP1 function has been found to lead to aberrant acquired mis‐splicing, a process that promotes cancer [101, 102]. Meanwhile, the most widespread role of PTBP1 is that it is a major repressive splicing factor involved in regulating variable splicing of hnRNAs, leading to exon skipping [20]. It was found that in human gliomas, PTBP1 promotes RTN4 hnRNA exon 3 jumping and reduces cell adhesion, thereby promoting cell migration and invasion [68]. In glioblastoma, PTBP1 binds to the hnRNA of USP5, leading to the jump of exon 15, which results in the generation of the short isoform USP5‐2, thus promoting the migration and invasion of tumour cells; by mutating the splice site of PTBP1, the expression of the USP5‐2 isoform can be inhibited, which reduces the ability of cells to migrate and invade [67]. In glioblastoma, renal clear cell carcinoma, bladder cancer, and colorectal cancer, PTBP1 promotes the conversion of PKM from PKM1 isoform to PKM2 isoform through inhibition of exon 10 of pyruvate kinase (PKM; Pyruvate Kinase M1/2) hnRNA, which facilitates the proliferation of tumour cells through the Warburg effect [62, 103]. In breast cancer, PTBP1 increases the expression of CDC42‐v1 isoform, which promotes the formation of filamentous pseudopods through variable splicing and improves tumour cells' migration and invasion ability [104]. Additionally, in hepatocellular carcinoma, PTBP1 regulates the cholesterol metabolism‐related genes HMGCR and LDLR through variable splicing to lower plasma cholesterol levels and cause abnormal lipid metabolism [105, 106]. PTBP1 forms the Axl‐S isoform by competitively binding to the intron 9 sequence of Axl precursor mRNA with U2AF2, leading to exon 10 jumps. The Axl‐S isoform encodes a protein that binds tightly to the Gas6 ligand, leading to phosphorylation of ERK and AKT proteins, which ultimately promotes migration and invasion of hepatocellular carcinoma cells [69]. In glioblastoma, PTBP1 functions as a splicing regulator by competitively binding to conserved sequences at multiple sites of BAF45d precursor mRNA. Two isoforms of BAF45d exist: the BAF45d (6A+) isoform, which is found in normal brain tissues, and the BAF45d (6A−) isoform, which is found only in glioblastomas. PTBP1 represses the expression of exon 6A and promotes the formation of the BAF45d (6A−) subtype [63] (Figure 3).

In human tumours, PTBP1 variable splicing abnormalities occur frequently, which may be due to mutations in splicing regulatory elements of specific cancer‐related genes or changes in splicing regulatory processes. Variable splicing also generates a new class of tumour proteins or tumour suppressors that promote disease progression by regulating RNA isoforms associated with tumour signalling pathways. Dysregulation of variable splicing is a fundamental process in cancer. Probing the regulators of the splicing machinery is a critical step in understanding the role of variable splicing in cancer and provides new targets and biomarkers for tumour therapy.

### 2.1 mRNA Stability

**FIGURE 4:**
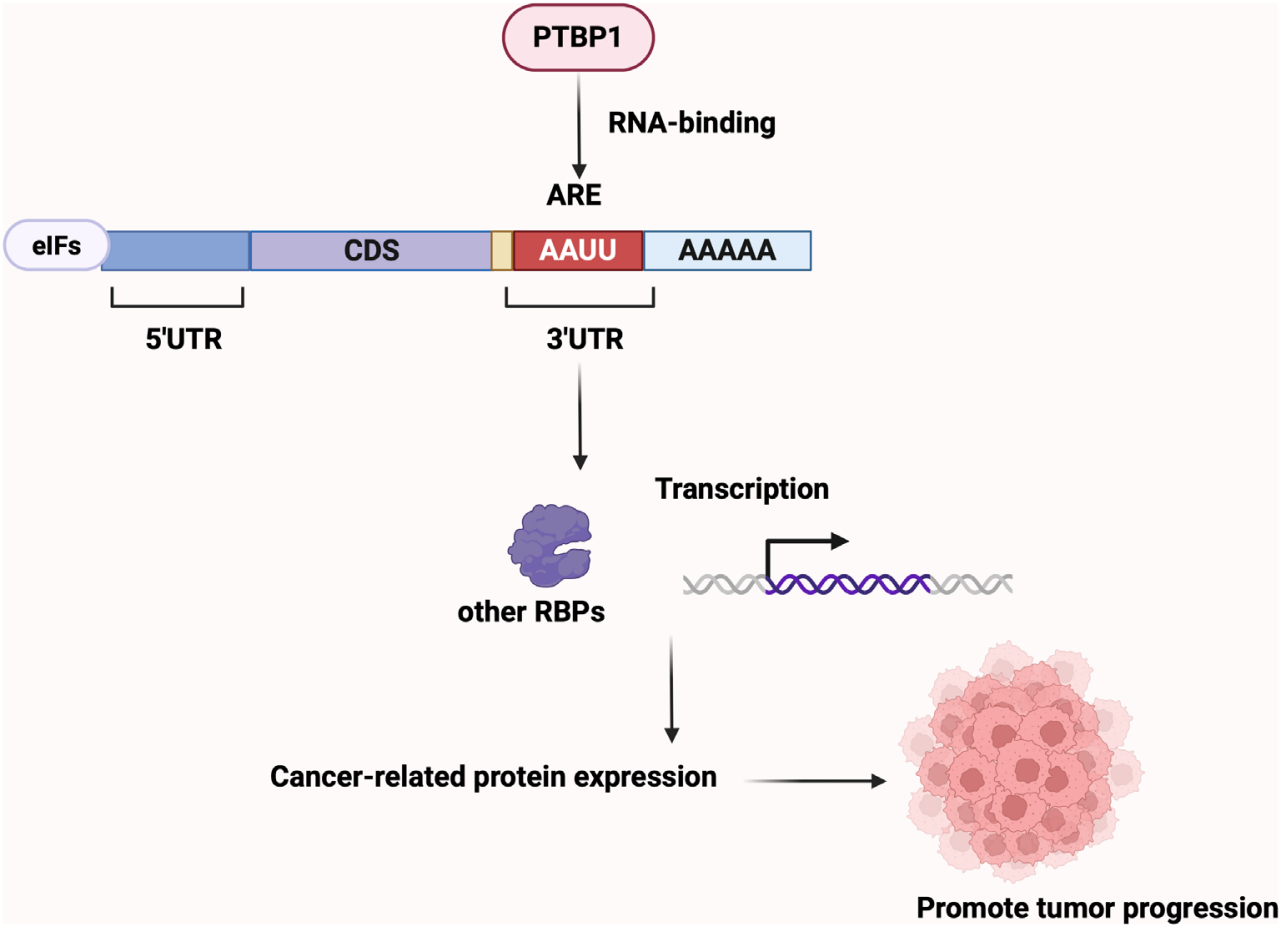
PTBP1 and ARE structure. The ARE structure is the most studied structure related to mRNA stabilisation. Abnormal increase and extension of the ARE mRNA encoding is associated with cancer progression. For example, PTBP1 enhances the stability of tumour‐associated mRNA by binding to the ARE structure of mRNA 3′ UTR, thereby increasing the expression of cancer‐associated proteins and promoting tumour angiogenesis, migration, invasion and drug resistance.

The stability of mRNAs is closely linked to RNA‐binding proteins (RBPs) and thus performs a variety of cellular functions. Moreover to the poly A tail at the 3′ end, structures affecting the stability of eukaryotic mRNAs include the cap structure at the 5′ end. mRNA degradation occurs in a variety of ways, either starting with hydrolysis of the poly A tail, followed by decapitation at the 5′ end and degradation in the 5′ → 3′ direction, or after hydrolysis of the poly‐A tail, degradation in the 3′ → 5′ direction [107]. To date, the structure involved in reverse mRNA degradation is the AU‐rich elements (ARE) 3′ UTR, and it has been estimated that 16% of transcripts contain ARE structures [108]. When cells are not stimulated, mRNAs containing ARE structures are degraded by poly(A) tails at all times, and ARE‐binding proteins (ARBPs) may promote mRNA degradation or enhance its stability after cells are differentially stimulated [109]. The ARE structure is the most studied structure related to mRNA stabilisation [110]. Abnormal increase and extension of coding ARE mRNAs are associated with cancer progression; for example, PTBP1 enhances the stability of tumour‐associated mRNAs by binding to the ARE structure in the 3′ UTR of mRNAs, which increases the expression of cancer‐associated proteins and promotes tumour angiogenesis, migration, invasion, and drug resistance (Figure 4) [111], suggesting that mRNA stability plays an important role. Additionally, in tumour tissues, PTBP1 binding to the 5′ UTR decreases mRNA stability. In lung adenocarcinoma, CL1 cells were isolated and cultured from tissues of patients with poorly differentiated adenocarcinoma. After subculture, CL1 cells were heterogenetically altered and gave birth to a more aggressive sublineage CL1‐5, which expressed more hypoxia inducible factor‐1 (HIF‐1) mRNA and protein than CL1 cells. In the CL1 cell line, PTBP1 can bind to the 5′ UTR of HIF‐1 mRNA, resulting in decreased stability of HIF‐1 mRNA, which is an important reason for the differential expression of HIF‐1 between the two sublines [45]. Also in lung cancer, it has been shown that PTBP1 binds to the 5′ UTR of the mRNA of the transmembrane receptor tyrosine kinase AXL causing a decrease in its stability and thus affecting tumour progression [59]. The above findings confirm that PTBP1 affects mRNA stability by binding to the 3′ UTR or 5′ UTR of mRNA.

### 2.2 mRNA Subcellular Localization

Some mRNAs are transcribed in the nucleus and then translocated to the cytoplasm and translated to produce proteins [112, 113]. However, other mRNA transcripts directly enter specific regions for localised translation or distribution, resulting in an asymmetric distribution of cytoplasmic proteins [114]. Subcellular localization of RNAs occurs mainly through RBP recognition of cis motifs or the formation of secondary structures by specific binding elements (zipcodes) in the 3′ UTR of target genes, which serve as binding sites for RBPs to regulates intracellular localization, which in turn mediates RNA localization to specific subcellular compartments [115, 116]. The process is that hnRNPs in the nucleus recognise and bind mRNAs, which are then transported to the cytoplasm, followed by the return of one part of the hnRNPs to the nucleus and the formation of a complex with motor proteins by the other part [114, 117]. This process is essential for maintaining cell polarity, and its dysregulation may lead to cancer progression [118].

PTBP1 regulates mRNA subcellular localization. It was found that in tumour cells, knockdown of PTBP1 caused MCL1 mRNA to accumulate in the cytoplasm in addition to increasing MCL1 mRNA levels, upregulating the cytoplasmic/nuclear ratio, but not decreasing the amount of mRNA in the nucleus [119]. In contrast, in CD4+ T cells, down‐regulation of PTBP1 increased the plasma/nucleus ratio of CD40L mRNA, resulting in a decrease in CD40L mRNA levels in the nucleus and an increase in cytoplasmic levels [120].

In summary, PTBP1 plays multiple roles in RNA cellular localization and ensures that RNAs can be correctly localized within cells by regulating multiple biological processes. Its role in different cell types and its relationship with cancer further emphasise the importance of PTBP1 in tumour biology. An in‐depth investigation of the function of PTBP1 will not only help to reveal the basic mechanism of RNA cellular localization, but also has the potential to provide new ideas and strategies for the treatment of related cancers.

### 2.3 mRNA Translation

The translation process is intricate and can be divided into three stages: initiation, extension and termination. Translational regulation of most mRNAs occurs at the initial stage [121]. RBPs, such as the 5′ cap‐binding complex eIF4F and poly A‐binding protein (PABP), are required for mRNA cyclization and translational activation [122]. In cancer, almost all key oncogenic signalling pathways are aberrant and cause translational deficits, such as the PI3K/AKT/mTOR, RAS/MAPK and Wnt/β‐catenin signalling pathways [122, 123, 124]. Dysregulated expression of eIF4E, a component of the eIF4F complex, has been reported to be associated with approximately 30% of human tumours [125]. Moreover, it has been shown that eIF4E knockout mice not only maintain normal physiology, but also have significant tumour suppression effects [126]. During cancer development, certain structure‐ and sequence‐specific regulatory genes can control protein translation. As one of these organising elements, the 5′ UTR has received much attention. 5′ UTR is an internal ribosome entry site (IRES) that recruits ribosomes directly in association with IRES transacting factors (ITAFs) to initiate translation in a cap‐dependent manner. Cancer development has been associated with dysregulation of this structure‐dependent translation [127, 128, 129]. It was found that PTBP1 acts as a transacting factor binding to the 5′ UTR of mRNA to upregulate the activity of mRNA IRES resulting in IRES‐dependent upregulation of translation levels. It has been shown that in HeLa cells, knockdown of PTBP1 reduced the activity of P27 mRNA IRES, resulting in decreased translation levels [130]. The level of the rhythm‐regulated gene PER1 is also regulated by PTBP1, which binds to IRES on the mRNA 5′ UTR of PER1 to positively regulate IRES‐mediated translation of PER1 without affecting PER1 mRNA levels [72]. In addition to affecting the post‐transcriptional level of proteins by regulating IRES activity, PTBP1 can also affect the post‐transcriptional level of proteins by directly binding to the mRNA 3′ UTR, and it has been found that in HeLa cells, PTBP1 binding to the 3′ UTR of HIF‐1α mRNA promotes HIF‐1α translation [131].

In summary, the role of PTBP1 in translation is multifaceted, involving the translation efficiency of mRNAs and the regulation of cell biological functions. By binding to specific regions of mRNAs, PTBP1 is able to effectively regulate protein synthesis and affect the functional and physiological state of cells. Future studies may reveal the deeper functions of PTBP1 in different physiological and pathological states and provide more clues to understand its importance in tumour biology.

### 2.4 The Role of Molecules Regulating PTBP1 in Cancer

**FIGURE 5:**
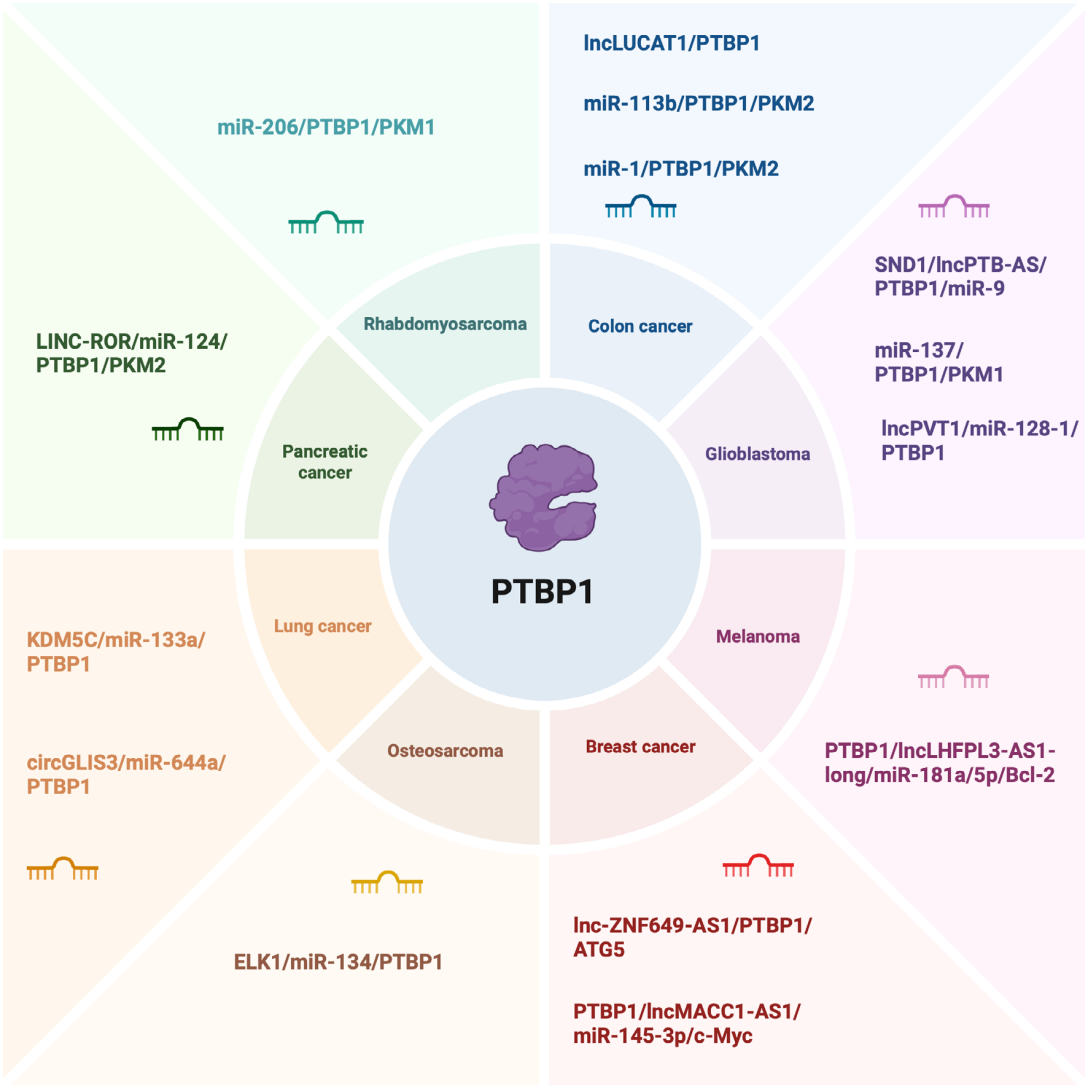
Regulatory relationship between PTBP1 and non‐coding RNA in multiple tumours.

Non‐coding RNA (ncRNA) is a class of RNAs that do not encode proteins and perform biological functions at the RNA level, and are involved in the regulation of transcription processes together with PTBP1. The more studied ncRNAs are long non‐coding RNA (lncRNA), micro RNA (miRNA) and circular RNA (circRNA) [132, 133]. More and more studies have demonstrated that non‐coding RNAs play a role in tumours, and it has been shown that PTBP1 binding to non‐coding RNAs would play a regulatory role in tumours [134]. Therefore, we summarised the biological functions exerted by PTBP1 interacting with several common non‐coding RNAs in different tumours (Figure 5).

Studies have shown that many non‐coding RNAs can bind to PTBP1 to influence the Warburg effect to affect tumour energy metabolism. In colon cancer, miR‐1 and miR‐133b expression is downregulated, and overexpression of miR‐1 and miR‐133b binds PTBP1 mRNA to reduce PTBP1 expression and inhibit the Warburg effect [135]. Brain‐specific miR‐137 and muscle‐specific miR‐206 regulate PKM expression and inhibit the Warburg effect by directly binding PTBP1 mRNA in glioblastoma and rhabdomyosarcoma, respectively [74]. In bladder cancer, miR‐145 interferes with the Warburg effect by inhibiting the KLF4/PTBP1/PKMs pathway in bladder cancer cells to suppress tumour growth [76]. Additionally, certain non‐coding RNAs produce drug resistance by directly binding to PTBP1. In colorectal cancer cells, hypoxia‐induced lncRNA LUCAT1 interacts with PTBP1 and promotes the binding of a series of DNA damage‐related genes to PTBP1, leading to alterations in the selective splicing of these genes and resulting in chemoresistance in colorectal cancer cells [136]. In pancreatic cancer cells, linc‐ROR regulation of the miR‐124/PTBP1/PKM2 axis of action resulted in gemcitabine resistance in pancreatic cancer cells [137]. In osteosarcoma cells, the ELK1/miR‐134/PTBP1 axis of action promotes resistance to adriamycin in osteosarcoma cells [138]. In breast cancer, lncRNA ZNF649‐AS1 induced trastuzumab resistance by increasing the expression level of ATG5 by enhancing the stability of ATG5 mRNA through binding to PTBP1 [139]. The presence of the blood–brain tumour barrier (BTB) restricts the transport of chemotherapeutic agents to brain tumour tissues and is an important obstacle to tumour therapy, especially glioma treatment. One study demonstrated that the interaction of lnc00462717 with PTBP1 inhibited the miR‐186‐5p/occludin pathway and reduced the permeability of the blood–brain tumour barrier [140]. PTBP1 can also affect the permeability of the blood–brain tumour barrier through the PTBP1/circRNA_001160/miR‐195‐5p/ETV1 axis of action [141]. Furthermore, in gliomas, lncRNA PVT1 binds specifically to miR‐128‐1‐5p, affects the expression of the miR‐128‐1‐5p target gene PTBP1, promotes apoptosis of glioma cells, and inhibits their proliferation [142]. In hepatocellular carcinoma, miR‐194 inhibits PTBP1 expression by binding to the 3′‐UTR of PTBP1 mRNA, leading to a decrease in cell cycle protein D3 (CCND3) protein levels, which affects the cell cycle and tumour progression [143]. In melanoma, PTBP1‐mediated splicing of the lncRNA LHFPL3‐AS1 precursor produces the lncRNA LHFPL3‐AS1‐long that inhibits the degradation of its target gene, Bcl‐2 mRNA, and upregulates the expression of Bcl‐2 by acting on miR‐181, which inhibits apoptosis and promotes melanoma stem cell death [144]. In addition, there are non‐coding RNAs that bind to PTBP1 to affect tumour progression through other ways. In gliomas, lncRNA ST7‐AS1 acts as a target gene of P53 and exerts tumour suppression by interacting with PTBP1 to inhibit the Wnt/β‐catenin signalling pathway [145]. lncRNA PTB‐AS binds to the 3′ UTR of PTBP1 and stabilises its mRNA to increase PTBP1 expression and promotes glioma progression. Furthermore, PTB‐AS can shield the binding site of miR‐9 in the 3′ UTR of PTBP1 and reduce the negative regulation of PTBP1 by miR‐9 [146]. In lung cancer cells, miR‐133a inhibits lung cancer growth and metastasis by binding to PTBP1 [147]. In non‐small cell lung cancer, the circular RNA circGLIS3 acts as a molecular sponge for miR‐644a and binds to miR‐644a to up‐regulate the expression of PTBP1, while PTBP1 binds to the flanking introns of circGLIS3 and promotes the cyclization of circGLIS3, forming a circGLIS3/miR‐644a/PTBP1 positive feedback loop that promotes the progression of non‐small cell lung cancer [85]. In bladder cancer, both lncHCG22 and lncMAFG‐AS1 can affect tumour progression through the HuR/PTBP1 axis. HuR, also known as ELAVL1, is an RNA‐binding protein, in which lncHCG22 inhibits bladder cancer progression by destabilising HuR proteins to regulate PTBP1 levels. In contrast, lncMAFG‐AS1 increases PTBP1 expression and promotes bladder uroepithelial cancer progression by decreasing ubiquitination degradation of HuR [148, 149]. In breast cancer cells, binding of lncRNA MACC1‐AS1 to PTBP1 reduces its own degradation and increases its sponge effect on multiple oncogenic miRNAs (miR‐145‐3p, miR‐384, or miR‐342‐5p) to promote cell proliferation and breast tumour progression. Furthermore, MACC1‐AS1 competes for PTBP1 with PTBP1 target genes and reduces the binding of PTBP1 to downstream target genes (e.g., MCL‐1 and PKM1) [150].

In summary, in tumour cells, PTBP1 interacts with a variety of non‐coding RNAs to affect tumour progression and therapy, suggesting that PTBP1 may be one of the key factors coordinating the role of non‐coding RNAs. However, the current context of PTBP1's role with various types of non‐coding RNAs is not clear, and studying the physiological basis affecting PTBP1's role with non‐coding RNAs may be the key to deciphering how non‐coding RNAs function in cancer.

### 2.5 PTBP1 and Tumour Therapy

Based on the important function of PTBP1 in cancer, there are small nucleic acid drugs and immunotherapy for its treatment in tumours, but the research for the above therapeutic methods is still less.

Inhibition of splicing factors by oligonucleotide (ON) method has been more studied in recent years [151]. Oligonucleotide is a general term for a class of short‐chain nucleotides with less than 50 bases. Oligonucleotides can easily bind to their complementary strands, so they are often used as probes for experiments such as gene chips, electrophoresis, and fluorescence in situ hybridization [152, 153]. Oligonucleotide drugs are a class of drugs consisting of artificially chemically synthesised single or double strands of 12–30 ribose oligonucleotides, which act on the target mRNA through the Watson‐Crick base pairing principle, and are mainly divided into two categories: antisense oligonucleotide (ASO) drugs and small interfering RNA (siRNA) drugs [154]. Antisense oligonucleotides are DNA fragments with a length of 10–20 bp, which can form heteroduplexes after entering cells by complementary binding with the mRNA of the genes they target, which not only prevents the binding of mRNA to ribosomes, but also activates ribonuclease H (RNase H) to cleave the heteroduplexes and degrade them, thus decreasing the content of mRNA of the target molecules [155, 156]. Hypoxia was found to induce the production of the lncRNA LUCAT1 in colorectal cancer, which promotes the growth of colorectal cancer cells and is associated with drug resistance of colorectal cancer cells in vitro and in vivo [157]. Ectopic expression of PTBP1 abrogated the effects induced by knockdown of LUCAT1, suggesting that LUCAT1 interacted with PTBP1 in colorectal cancer cells and promoted the AS of DNA damage‐related genes. Therefore, ASO drugs designed to target LUCAT1 can disrupt the LUCAT1‐PTBP1 axis, thereby inhibiting the proliferation of colorectal cancer cells and decreasing cellular resistance to DNA‐damaging drugs [136, 158]. siRNAs are double‐stranded RNAs that are 20–25 nucleotides long, and are mainly involved in the phenomenon of RNA interference in biology [159]. The pro‐invasive effects of PTBP1 can be significantly reduced by using siRNA targeting PTBP1. For example, the pro‐invasive role of PTBP1 in bladder carcinogenesis can also be inhibited to some extent by in vivo injection of siRNA [160]. Additionally, combined small interfering RNA and tiny RNA treatments to modulate tumour‐specific energy metabolism of driver oncogenes is a novel form of interference. For example, mRNA and protein expression of the transcription factors c‐Myc and PTBP1 were reduced after miR‐145 and siR‐PTBP1 co‐treatment [89].

ASO and siRNA may be toxic to cells [161], so some studies have designed decoy oligonucleotides that bind specifically to splicing factors and exert inhibitory effects on splicing processes and biological activities in vitro and in vivo [162]. Decoy oligonucleotides targeting PTBP1, when introduced into cells, are able to accurately and directly bind to each other and to the PTBP1 protein, interrupting its binding to hnRNA and thus inhibiting the role of PTBP1 in tumours [160]. For example, the introduction of PTBP1‐baiting oligonucleotides into glioblastoma cells resulted in suppression of intracellular expression of multiple oncogenic protein isoforms, resulting in reduced cell proliferation, clone formation, and tumorigenicity [160].

Immunity plays an important role in the treatment of tumours [163, 164]. Studies have found that among 30 tumour types, the positive correlation between PTBP1 expression and LAG3 and PD‐1 expression is the most common [165]. Studies have found that the specific defect of PTBP1 in dendritic cells can increase the expression of major histocompatibility complex II and disrupt T cell homeostasis, but it does not affect the differentiation and development of dendritic cells themselves. PTBP1 mainly regulates the selective splicing of muscular pyruvate kinase and participates in the expression of interferon‐related downstream signalling pathways. Therefore, targeting PTBP1 in dendritic cells can promote anti‐tumour immunity [165]. The PTBP family was further investigated by comparing PTBP expression in paired and unpaired tissue samples from the Tumour Genome Atlas database. PTBP was found to be widely upregulated in human tumour tissues and, significantly differentially expressed in tumour immune subtypes, and strongly correlated with tumour‐infiltrating lymphocytes in the tumour microenvironment [165]. Gene enrichment analysis showed that the PTBP family was associated with the processes of gene‐selective splicing, cellular senescence, and protein modification in tumour cells, confirming the application of PTBP as a potential tumour immunotherapy target [166].

In conclusion, the current clinical application studies for PTBP1 are now relatively few, and there are no more clinical trial data to support the translation of PTBP1 inhibitors for clinical application. At present, the mini‐nucleic acid drug can only be used as an adjuvant chemotherapy due to its safety, and further clinical application requires more research. In conclusion, PTBP1, a splicing factor, is involved in a wide range of cellular biological processes, and research on PTBP1 is expected to find new ideas for treating cancer and other diseases.

## 3 Discussion

PTBP1, as an RNA‐binding protein, is involved in regulating various intracellular biological processes such as splicing, translation, stability, and localization of mRNAs and is extremely closely related to tumours. However, research on PTBP1 is currently facing many difficulties and challenges, and we summarise the following points regarding the difficulties and challenges encountered in our current research: first, the current technologies used to study the structure and function of PTBP1 are still limited. Understanding its interaction with RNA requires high‐resolution structural biology techniques, but the application of these techniques to the intracellular environment remains challenging. Second, PTBP1 may exhibit different functions in different cell types and physiological situations, which complicates the study of its generalisation and specificity. Different experimental models may lead to a different understanding of PTBP1 function. Finally, the aberrant expression of PTBP1 has been associated with a wide range of cancers, but the exact mechanisms have not been fully elucidated. This complexity makes it challenging for researchers to explore PTBP1 as a potential therapeutic target.

The current study suggests that research on the role of PTBP1 in cancer has excellent potential for clinical translation. We summarise the clinical translational potential of PTBP1 research as follows: potential therapeutic targets due to the critical role of PTBP1 in the progression of various cancers; factors and non‐coding RNAs related to its regulatory process have become new potential therapeutic targets. By inhibiting specific factors and non‐coding RNAs, the survival signals of tumour cells can be targeted to intervene, thus improving the therapeutic effect. To promote drug development, PTBP1 is closely related to tumour progression and involved in the alteration of relevant pathway activities in tumour therapy, including the targets of many clinical drugs that have been widely used. However, it is still unknown whether antagonising PTBP1 in clinical treatment can improve drug therapeutic efficacy and whether drug development targeting PTBP1 can improve patients' prognosis, and it would significantly improve tumour therapeutic efficacy if the impact of PTBP1 in different tumour drug therapies can be clarified. Potential as a biomarker, the status of PTBP1 may serve as a biomarker of prognosis and response to treatment in cancer patients, helping to assess patient response to treatment and guiding clinical decision‐making.

In summary, based on the critical function of PTBP1 in cancer and the difficulties and challenges in its study, future research should focus on the following aspects: an in‐depth analysis of the structure–function relationship of PTBP1: resolving the structure of PTBP1 in complex with RNA and other proteins by techniques such as X‐ray crystallography or cryo‐electron microscopy will help to understand its specific functional mechanism. Investigating the role of PTBP1 in different biological processes (e.g., development, regeneration, and disease genesis), especially its potential role in cancer, will help reveal its importance in regulating cellular functions. Given the relevance of PTBP1 in many cancers, future studies could focus on developing drugs or therapeutic strategies targeting PTBP1 to modulate its function and improve tumour therapeutic efficacy, which may include small molecule inhibitors or RNA interference technologies, among others. Studying the interaction network of PTBP1 with other proteins and RNAs through a systems biology approach will help to understand its multiple roles within the cell and how these interactions are regulated under different physiological and pathological conditions.

In conclusion, PTBP1 is a multifunctional protein with critical biological functions, and the relationship between its structure and function still requires in‐depth study. Through a comprehensive understanding of its structure, function, and interactions, future studies can reveal PTBP1's key role in cell biology and provide new ideas for the treatment of related diseases. With continuous research, PTBP1 may become an essential target in biomedical research.

## Author Contributions

**Chengdong Ji:** conceptualization (equal), data curation (equal), project administration (equal), software (equal), writing – original draft (equal). **Jing Zhu:** investigation (equal), writing – original draft (equal). **Xiaochen Hou:** investigation (equal), supervision (equal), validation (equal), writing – original draft (equal). **Chen Zhou:** formal analysis (equal), validation (equal). **Jiumei Zhao:** data curation (equal), software (equal). **Xiang Zheng:** data curation (equal), formal analysis (equal), software (equal). **Yu Tang:** conceptualization (equal), data curation (equal), formal analysis (equal), funding acquisition (equal), writing – original draft (equal), writing – review and editing (equal).

## Ethics Statement

The authors have nothing to report.

## Consent

The authors have nothing to report.

## Conflicts of Interest

The authors declare no conflicts of interest.

## Data Availability

All references are from the PubMed public database with authentic and usable data.
